# Effect of *Cyberlindnera jadinii* supplementation on growth performance, serum immunity, antioxidant status, and intestinal health in winter fur-growing raccoon dogs (*Nyctereutes procyonoides*)

**DOI:** 10.3389/fvets.2023.1154808

**Published:** 2023-05-11

**Authors:** Dehui Zhao, Haihua Zhang, Keyuan Liu, Yan Wu, Borui Zhang, Cuiliu Ma, Hanlu Liu

**Affiliations:** ^1^College of Agriculture, Chifeng University, Chifeng, China; ^2^Hebei Key Laboratory of Specialty Animal Germplasm Resources Exploration and Innovation, Hebei Normal University of Science and Technology, Qinhuangdao, Hebei, China; ^3^Institute of Special Animal and Plant Sciences of Chinese Academy of Agricultural Sciences, Changchun, China; ^4^College of Animal Science and Technology, Qingdao Agricultural University, Qingdao, China

**Keywords:** *Cyberlindnera jadinii*, growth performance, immunity, antioxidant, microbiota, raccoon dogs

## Abstract

**Introduction:**

This study aimed to investigate the effects of *Cyberlindnera jadinii* supplementation on the growth performance, nutrient utilization, serum biochemistry, immunity, antioxidant status, and intestinal microbiota of raccoon dogs during the winter fur-growing period.

**Methods:**

Forty-five 135 (±5) day-old male raccoon dogs were randomly assigned to three dietary groups supplemented with 0 (group N), 1 × 10^9^ (group L) and 5 × 10^9^ CFU/g (group H) *Cyberlindnera jadinii*, with 15 raccoon dogs per group.

**Results:**

The results showed that *Cyberlindnera jadinii* in groups L and H improved average daily gain (ADG) and decreased feed-to-weight ratio (F/G) (*P* < 0.05). No significant difference was found in nutrient digestibility and nitrogen metabolism among the three groups (*P* > 0.05). Compared with group N, serum glucose levels were lower in groups L and H (*P* < 0.05). The levels of serum immunoglobulins A and G in group L were higher than those in the other two groups (*P* < 0.05), and the levels of serum immunoglobulins A and M in group H were higher than those in group N (*P* < 0.05). Supplementation with *Cyberlindnera jadinii* in groups L and H increased serum superoxide dismutase activity, and the total antioxidant capacity in group H increased compared with group N (*P* < 0.05). The phyla Bacteroidetes and Firmicutes were dominant in raccoon dogs. The results of principal coordinate analysis (PCoA) showed that the composition of microbiota in the three groups changed significantly (*P* < 0.05). The relative abundance of Campylobacterota was increased in the H group compared to the N and L groups (*P* < 0.05). The relative abundance of *Sarcina* was increased in group L compared with the other two groups (*P* < 0.05), while the relative abundance of *Subdoligranulum* and *Blautia* were decreased in group H compared with the other two groups (*P* < 0.05). Also, the relative abundance of *Prevotella, Sutterella* and *Catenibacterium* was higher in group L (*P* < 0.05) compared with group H.

**Discussion:**

In conclusion, dietary supplementation with *Cyberlindnera jadinii* improved growth performance, antioxidant activity, immune status, and improved intestinal microbiota in winter fur-growing raccoon dogs. Among the concentrations tested, 1 × 10^9^ CFU/g was the most effective level of supplementation.

## Introduction

*Cyberlindnera jadinii*, an anamorphic form of *Candida utilis*, is used in the food and fodder industries (1, 2). *Cyberlindnera jadinii* is capable of producing valuable bioproducts and is an attractive source of biomass rich in protein and vitamins (3). It has been reported that *Cyberlindnera jadinii* has potential as an unconventional protein feed ingredients (4) and can replace 10% of soybean and meal crude protein in broiler chicken diets while maintaining growth performance and digestive function (5). More importantly, as a feed additive, *Cyberlindnera jadinii* can also improve animal health, promote growth and development, and improve feed efficiency. For example, the dietary supplementation with *Cyberlindnera jadinii* could not only improve the ruminal ammonia nitrogen contents of total volatile fatty acids and propionic acid of dairy cows and reduce the ruminal acetic acid concentration and acetic acid to propionic acid ratio, but also has a tendency to increase feed efficiency (6). Dietary supplementation with *Cyberlindnera jadinii* also improved feed conversion ratio and protein digestibility in Hu sheep (7), which was found to improve gut homeostasis and increase cecal microbial diversity in weaned piglets (8, 9). Recent studies demonstrated that dietary supplementation with *Cyberlindnera jadinii* increases feed intake of fodder in total mixed ratio and carcass weight in beef cattle aged 15 months, and also decrease dry matter intake and feed to weight ratio (10). However, no research data on *Cyberlindnera jadinii* in the diet of canine animals was available.

The Ussuri raccoon dog is a very ancient canid with a short, fat body somewhere between a raccoon and a dog. Besides, the Ussuri raccoon dog is the only hibernating animal among the canids, and its utilization of nutrients has its own unique characteristics, such as the demand for higher animal protein ingredients than that of red foxes, and it has certain requirements for fruits and grains in the diets. The main function of the gut is to digest food and absorb nutrients (11), and the gut microbiota is involved in the digestion and absorption of nutrients, maturation and regulation of the immune system (12, 13). In our previous study, we have found that supplementation with 1 × 10^9^ CFU/g *Cyberlindnera jadinii* has a positive impact on growth performance and intestinal microbiota in growing raccoon dogs (14). However, the winter fur-growing period is a key stage for the growth and development of raccoon dogs, and a key stage for producing high economic value. Yet, the effect of *Cyberlindnera jadinii* on the winter fur-growing period of raccoon dog has not been studied to date. We hypothesized that *Cyberlindnera jadinii* might affect the growth and gut microbiota of winter fur-growing raccoon dogs. Therefore, the objectives of the present research were to study the effects of *Cyberlindnera jadinii* on the growth performance, nutrient digestibility, nitrogen metabolism, serum biochemical parameters, antioxidant capacity and gut microbiota of winter fur-growing raccoon dog. Our results may help to better apply *Cyberlindnera jadinii* to the production of raccoon dog and the development of canine products.

## Materials and methods

All procedures involving animals were carried out in accordance with the guidelines for animal studies issued by Chifeng University.

### Fungal strain

*Cyberlindnera jadinii* (Number: YTCJ91011) was preserved by the microbiology laboratory of the Chifeng University. Liquid potato dextrose culture medium (PDB) (Potato extract powder 20 g/L, glucose 20 g/L, distilled water 1 L) was used for resuscitating and subcultures (aerobic cultivation) at 28°C for 24 h.

### Animal husbandry and experimental design

Forty-five healthy male raccoon dogs aged 135 ± 5 days and with similar body weight (7.78 ± 0.62 kg) were randomly assigned to three treatment groups, with 15 replicates per group. All animals were obtained from Shenyang Boyang Feed Co., Ltd. The *Cyberlindnera jadinii* was either not supplemented in the diet (group N) or supplemented with 1 × 10^9^ CFU/g (group L) or 5 × 10^9^ CFU/g (group H). The dosing basis for *Cyberlindnera jadinii* was based on the study in weaned piglets (15). The basal diet was formulated according to the management guidelines of the National Research Council (NRC, 1982) (16), and the composition and nutrient levels of the basal diet are shown in Table 1. All animals were housed individually in conventional cages (1.0 × 0.8 × 0.8 m). Raccoon dogs were fed twice daily at 7:00 AM and 15:00 PM with free access to water. After 7 days of adaptation, the experiment was lasted for 30 days.

**Table 1:** Composition and nutrient levels of basal diets (air-dry basis).

**Items**	**Content (%)**	**Items**	**Content (%)**
**Ingredients**		**Nutrient levels^b^**	
Extruded corn	42.81	Metabolizable energy (MJ/kg)^b^	14.78
Wheat bran	8.25	Crude protein	23.02
Corn germ meal	7.74	Ether extract	10.08
Rice bran meal	1.55	Ash	7.65
Soybean oil	7.22	Calcium	1.03
Soybean meal	10.83	Phosphorus	0.78
Distillers dried grains with solubles	6.71	Lysine	1.38
Fish meal	6.19	Methionine	0.95
Meat and bone meal	6.19	Methionine + Cysteine	1.22
Pork plasma protein powder	0.52		
NaCl	0.15		
Lysine	0.52		
Methionine	0.62		
Choline	0.08		
Premix^a^	0.62		
Total	100.00		

^a^The premix contains the following ingredients per kg of diet: VA 15 902 IU, VB_1_ 44 mg, VB_2_ 19 mg, VB_6_ 19 mg, VB_12_ 0.06 mg, VD_3_ 2 224 IU, VE 126 mg, VK_3_ 2 mg, biotin 0.55 mg, folic acid 1.0 mg, D-pantothenic acid 17 mg, nicotinamide 38 mg, antioxidant 0.5 mg, Cu (as copper sulfate) 32 mg, Fe (as ferrous sulfate) 118 mg, Mn (as manganese sulfate) 62 mg, Zn (as zinc sulfate) 100 mg, I (as calcium iodate) 0.31 mg, Se (as sodium selenite) 0.23 mg.

^b^Metabolizable energy was calculated based on the concentrations of crude protein, fat and carbohydrates according to from NRC (16), while others are measured values.

### Experimental procedures and sample collection

The raccoon dogs were weighed before morning feeding at the beginning and end of the experiment, and the animals' initial body weight (IBW) and final body weight (FBW) were used to determine average daily gain (ADG). On the 30th day of the experiment, eight raccoon dogs with similar weight and good health were randomly selected in each group for digestion and metabolism experiments. Urine and feces were collected for three consecutive days. At the end of the experiment, the urine was preserved with 10% sulfuric acid. The total volume amount of urine from each animal was recorded and filtered through filter paper. 10 mL of each filtrate were stored at −20°C. 10% sulfuric acid was added to feces in accordance with 5% of the fresh weight. All feces from one animal were mixed and weighted. 10% of the total weight of feces was dried at 65°C to constant weight, and then ground through a 40-mesh screen. The daily amount of feed provided and the amount of remaining feed during the experimental period were recorded. Meanwhile, 5 mL of blood samples from the veins of lower limbs of three groups of raccoon dogs were collected, placed in a serum separation tubes (BD-Pharmingen, USA), and centrifuged at 3,000 × g for 10 min to obtain serum. All serum samples were stored at −20°C for later analysis.

### Chemical analysis

Diets and feces were analyzed for dry matter (DM), ether extract (EE), crude protein (CP), ash (17). Dietary calcium and phosphorus were estimated by the AOAC method (17). Nitrogen in urine was analyzed according to the procedures of AOAC (17). All chemical analyses were conducted in triplicate.

### Serum sample analysis

Concentrations of serum total protein (TP), albumin (ALB), glucose (GLU), triglyceride (TG), highly density lipoprotein (HDL), low-density lipoprotein (LDL), cholesterol (CHO) and enzymatic activities of alkaline phosphatase (ALP), alanine aminotransferase (ALT) and aspartate aminotransferase (AST) (kits supplied by Zhongsheng Beikong Biotechnology Co., Ltd., Beijing, China) were analyzed by an automatic biochemical analyzer (Selectra-E, Witu). Serum immunoglobulins IgA, IgM, and IgG were measured using ELISA kits (Shanghai Shuangying Biotechnology Co., Ltd., Shanghai, China). The contents of glutathione peroxidase (GSH-Px), superoxide dismutase (SOD), total antioxidant capacity (T-AOC) and maleic dialdehyde (MDA) were determined using respective diagnostic kits (Nanjing Jiancheng Bioengineering Institute, Nanjing, China).

### DNA extraction, amplification, sequencing and bioinformatics analysis

Total bacterial DNA was extracted from the samples using the Qiagen magnetic bead extraction Kit (Qiagen, Valencia, California, USA) according to the manufacturer's instructions. Primers 341F (5′-CCTAYGGGRBGCASCAG-3′) and 806R (5′-GGACTACNNGGGTATCTAAT-3′) were used to amplify the V3–V4 region of the bacterial 16S rRNA gene. The resultant amplicons were purified using the Thermo Scientific GeneJET Gel Extraction Kit (Thermo Scientific, Belmont, Massachusetts, USA), and then sequenced on an Illumina NovaSeq 6000 platform to produce 250-bp paired-end reads (14).

Paired-end reads were merged into raw tags using FLASH (version 1.2.7) (18). Quality filtering (< 30 Phred score) of the raw tags was strictly performed with QIIME (version 1.9.1) to obtain high-quality clean tags (19, 20), which were compared to the SILVA database (version 138) using UCHIME algorithm to identify and remove chimeras of valid tags (21, 22). The Uparse algorithm (Uparse version 7.0.1001) was used to cluster valid tags from all samples and cluster sequences into operational taxonomic units (OTUs) with 97% identity (23). The sequence with the highest frequency of occurrence in OTUs was screened for further annotation. All analyses from clustering to determining alpha and beta diversity were performed with QIIME (version 1.9.1). The Adonis function of the R vegan package (version 2.15.3) was used to test the significance of separation by permutation multivariate analysis of variance (PERMANOVA) (24).

### Statistical analysis

The growth performance of each animal was calculated using the following formulas: ADG = (FBW– IBW)/30 days; Average daily feed intake (ADFI) = sum of daily feed intake/30 days; Feed conversion ratio (F:G) = ADFI/ADG.

Nitrogen content in diets, feces, and urine were calculated as crude protein (CP)/6.25. The nutrient apparent digestibility and nitrogen metabolism were calculated as follows: Nutrient apparent digestibility (g/Kg) = (feed nutrient intake – nutrient excretion in feces)/feed nutrient intake × 1000; Nitrogen retention = nitrogen intake – fecal nitrogen – urinary nitrogen; Net protein utilization (NPU) (%) = nitrogen retention/nitrogen intake × 100%; and Biological value of protein (BV) (%) = nitrogen retention/(nitrogen intake – fecal nitrogen) × 100%.

Data were analyzed by One-way analysis of variance (ANOVA) and Bonferroni multiple comparison test if the data were in Gaussian distribution and had equal variance or analyzed by the Kruskal-Wallis test and Bonferroni multiple comparison test if the data were not normally distributed. Data were represented as mean ± standard error. *P* < 0.05 indicates the difference is significant. STAMP software (*t*-test) was used to analyze the differences in the abundance of microbiota in each group, and the Benjamini-Hochberg FDR multiple test correction method was used to control the false positive rate.

## Results

### Growth performance

As shown in Table 2, there was no significant difference in IBW, FBW, or ADFI among the three groups (*P* > 0.05). ADG was increased in groups L and H compared to group N (*P* < 0.05), while F/G was decreased in groups L and H compared to group N (*P* < 0.05).

**Table 2:** Effects of *Cyberlindnera jadinii* on the growth performance of winter fur-growing raccoon dogs.

**Items**	**Group**			***P*-value**
	**N**	**L**	**H**	
IBW, kg	7.87 ± 0.17	7.75 ± 0.27	7.71 ± 0.24	0.868
FBW, kg	8.72 ± 0.11	9.04 ± 0.23	9.02 ± 0.26	0.495
ADG, g/d	31.48 ± 3.54^b^	48.05 ± 2.68^a^	48.52 ± 2.42^a^	0.001
ADFI, g/d	606.31 ± 8.96	594.79 ± 6.23	600.21 ± 10.11	0.644
F/G	20.92 ± 2.23^a^	12.58 ± 0.54^b^	12.54 ± 0.52^b^	< 0.001

IBW, Initial body weight; FBW, Final body weight; ADG, Average daily gain; ADFI, average daily feed intake; F/G, ratio of feed to gain. N group, 0 CFU/g *Cyberlindnera jadinii*; L group, 1 × 10^9^ CFU/g *Cyberlindnera jadinii*; H group, 5 × 10^9^ CFU/g *Cyberlindnera jadinii*. Data in the same row with no letter or with the same superscripts means no significant difference (*P* > 0.05); different lowercase letters mean significant difference (*P* < 0.05).

### Nutrients digestibility and nitrogen metabolism

There were no significant differences among the three groups in dry matter, ether extract and crude protein digestibility (*P* > 0.05, Table 3). There was also no difference in nitrogen intake, fecal nitrogen, urine nitrogen, nitrogen retention, NPU and BV (*P* > 0.05, Table 4) among the three groups.

**Table 3:** Effect of *Cyberlindnera jadinii* on nutrients digestibility of winter fur-growing raccoon dogs.

**Items**	**Group**			***P*-value**
	**N**	**L**	**H**	
Dry matter, %	85.80 ± 0.53	85.75 ± 1.71	85.71 ± 0.74	0.999
Ether extract, %	92.63 ± 0.82	91.24 ± 0.93	92.40 ± 0.88	0.500
Crude protein, %	85.61 ± 0.60	85.65 ± 1.71	85.74 ± 0.73	0.996

N group, 0 CFU/g *Cyberlindnera jadinii*; L group, 1 × 10^9^ CFU/g *Cyberlindnera jadinii*; H group, 5 × 10^9^ CFU/g *Cyberlindnera jadinii*. In the same row, values with no letter or with the same lowerletter superscripts indicate no significant difference (*P* > 0.05); superscripts with different lowercase letters indicate significant difference (*P* < 0.05).

**Table 4:** Effect of *Cyberlindnera jadinii* on N metabolism of winter fur-growing raccoon dogs.

**Items**	**Group**			***P*-value**
	**N**	**L**	**H**	
Nitrogen intake, g/d	20.71 ± 0.31	20.32 ± 0.21	20.50 ± 0.35	0.642
Fecal nitrogen, g/d	2.99 ± 0.16	2.91 ± 0.34	2.92 ± 0.16	0.967
Urine nitrogen, g/d	5.22 ± 0.40	5.46 ± 0.52	4.87 ± 0.33	0.611
Nitrogen retention, g/d	12.50 ± 0.45	11.94 ± 0.70	12.71 ± 0.47	0.602
NPU, %	60.45 ± 2.21	58.75 ± 3.27	62.01 ± 2.02	0.671
BV, %	70.53 ± 2.24	68.46 ± 3.10	72.25 ± 1.91	0.563

NPU, net protein utilization; BV, biological value of protein. N group, 0 CFU/g *Cyberlindnera jadinii*; L group, 1 × 10^9^ CFU/g *Cyberlindnera jadinii*; H group, 5 × 10^9^ CFU/g *Cyberlindnera jadinii*. In the same row, values with no letter or the same letter superscripts indicate no significant difference (*P* > 0.05); different small letter superscripts indicate a significant difference (*P* < 0.05).

### Serum biochemical parameters, immune levels, and antioxidant capacity of the three groups

As shown in Table 5, compared with group N, the serum GLU levels of groups L and H were decreased (*P* < 0.05), while the serum IgA levels of groups L and H was increased compared with group N (*P* < 0.05) as shown in Table 6, and the serum IgG levels was increased in group L compared with groups N and H (*P* < 0.05). The serum IgM levels was increased in group H compared with groups N and L (*P* < 0.05). The SOD activity in groups L and H was higher than that in group N (*P* < 0.05), but no significant difference was observed between groups L and H (*P* > 0.05). Serum T-AOC was increased in group H compared with groups N and L (*P* < 0.05), but no significant difference in serum MDA and GSH-Px activities were observed among the three groups (*P* > 0.05).

**Table 5:** Effects of *Cyberlindnera jadinii* on serum biochemical indices in winter fur-growing raccoon dogs.

**Items**	**Group**			***P*-value**
	**N**	**L**	**H**	
TP, mmol/L	80.15 ± 3.27	74.38 ± 2.07	81.37 ± 4.19	0.297
ALB, g/L	34.29 ± 1.27	31.43 ± 0.94	30.83 ± 2.87	0.401
GLU, mmol/L	3.72 ± 0.06^a^	3.24 ± 0.11^b^	2.89 ± 0.12^b^	< 0.001
ALP, U/L	29.96 ± 2.28	29.69 ± 1.45	29.84 ± 3.25	0.997
AST, U/L	55.17 ± 5.78	60.95 ± 2.12	65.13 ± 5.58	0.356
ALT, U/L	78.98 ± 14.29	57.21 ± 7.32	75.61 ± 15.24	0.432
TG, mmol/L	0.99 ± 0.14	0.75 ± 0.12	1.05 ± 0.13	0.264
CHO, mmol/L	4.25 ± 0.36	3.73 ± 0.34	3.31 ± 0.21	0.145
HDL, mmol/L	2.97 ± 0.24	2.62 ± 0.24	2.36 ± 0.11	0.135
LDL, mmol/L	0.25 ± 0.02	0.24 ± 0.03	0.21 ± 0.02	0.522

TP, totalprotein; ALB, albumin; GLU, glucose; ALP, alkaline phosphatase; AST, aspartate ami-notransferase; ALT, alanine aminotransferase; TG, triglycerides; CHO, cholesterol; HDL, highly density lipoprotein; LDL, low density lipoprotein. N group, 0 CFU/g *Cyberlindnera jadinii*; L group, 1 × 10^9^ CFU/g *Cyberlindnera jadinii*; H group, 5 × 10^9^ CFU/g *Cyberlindnera jadinii*. In the same row, values with no letter or the same letter superscripts indicate no significant difference (*P* > 0.05); different small letter superscripts indicate a significant difference (*P* < 0.05).

**Table 6:** Effects of *Cyberlindnera jadinii* on serum immune and antioxidant indices in winter fur-growing raccoon dogs.

**Items**	**Group**			***P*-value**
	**N**	**L**	**H**	
IgA (μg/ml)	40.31 ± 0.62^b^	44.58 ± 1.08^a^	44.43 ± 0.64^a^	0.003
IgG (μg/ml)	355.44 ± 4.96^b^	401.86 ± 6.97^a^	373.08 ± 8.26^b^	< 0.001
IgM (μg/ml)	18.40 ± 0.27^b^	18.22 ± 0.31^b^	20.87 ± 0.38^a^	< 0.001
GSH-Px, U/mL	1,011.11 ± 90.56	1,055.56 ± 74.14	1,025.93 ± 81.88	0.928
SOD, U/mL	17.03 ± 0.86^b^	19.58 ± 0.56^a^	20.32 ± 0.37^a^	0.005
T-AOC, U/mL	2.37 ± 0.25^b^	2.73 ± 0.12^ab^	3.26 ± 0.21^a^	0.020
MDA, nmol/mL	9.12 ± 0.39	9.66 ± 0.99	10.34 ± 1.87	0.786

GSH-Px, Glutathione peroxidase; SOD, Superoxide dismutase; T-AOC, Total antioxidant capacity; MDA, Malondialdehyde. N group, 0 CFU/g *Cyberlindnera jadinii*; L group, 1 × 10^9^ CFU/g *Cyberlindnera jadinii*; H group, 5 × 10^9^ CFU/g *Cyberlindnera jadinii*. In the same row, values with no letter or the same letter superscripts indicate no significant difference (*P* > 0.05); different small letter superscripts indicate a significant difference (*P* < 0.05).

### Summary of high-throughput sequencing and alpha diversity

**Figure 1:**
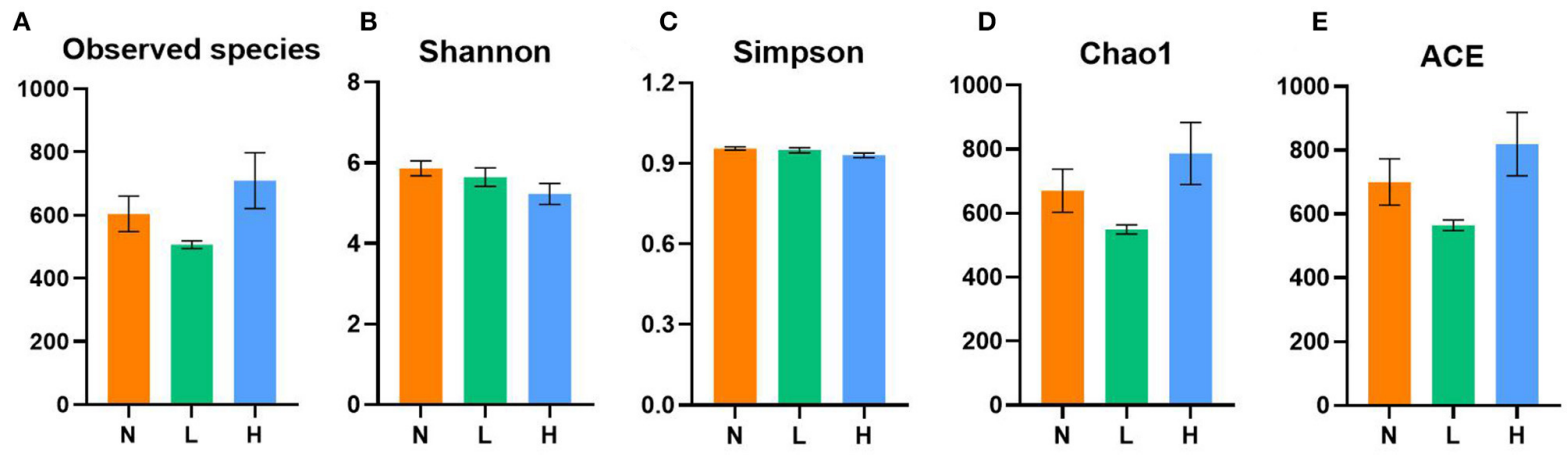
Comparisons of the alpha diversity indices of gut microbiota among the three groups of the raccoon dogs. Observed species **(A)**, Shannon index **(B)**, Simpson index **(C)**, Chao1 index **(D)**, and ACE index **(E)**. N group, 0 CFU/g *Cyberlindnera jadinii*; L group, 1 × 10^9^ CFU/g *Cyberlindnera jadinii*; H group, 5 × 10^9^ CFU/g *Cyberlindnera jadinii*.

A total of 1,572,834 16S rRNA gene sequences from the three designed groups were obtained from this study. After clustering at the 97% similarity level, sequences were assigned to 3,095 OTUs. Good coverage ranged from 0.996 to 0.999 demonstrated sufficient sequencing depth for all samples. As shown in Figure 1, no difference in the observed species, Shannon, Simpson, Chao1 and ACE indices was observed among the three groups (*P* > 0.05).

### Composition and comparison of gut microbiota

**Figure 2:**
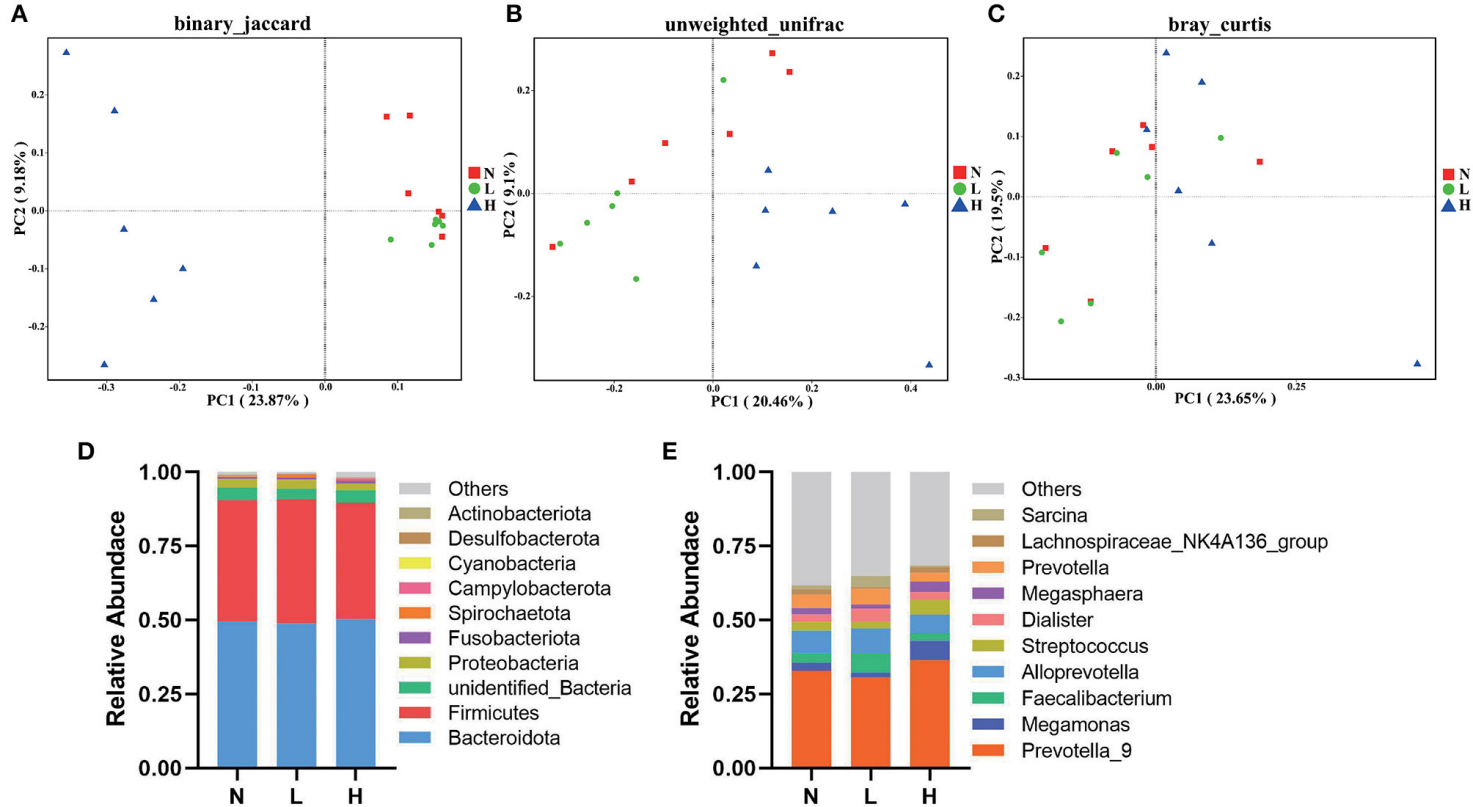
Composition and comparison of gut microbiota in three groups of raccoon dogs. PCoA revealed the separation of gut microbiota in the three groups based on binary Jaccard **(A)**, unweighted UniFrac distance **(B)**, and Bray–Curtis distance **(C)**. Gut microbial composition of the N, L and H group raccoon dogs at phylum **(D)** and genus **(E)** levels. N group, 0 CFU/g *Cyberlindnera jadinii*; L group, 1 × 10^9^ CFU/g *Cyberlindnera jadinii*; H group, 5 × 10^9^ CFU/g *Cyberlindnera jadinii*.

PCoA was applied to examine differences in taxonomic community composition and structure in the gut of raccoon dogs. PCoA based on binary Jaccard distance (Figure 2A) showed that group N was separated from group L [Table 7, Adonis: *P* < 0.05 (N vs. L)]. PCoA based on binary Jaccard distance (Figure 2A) and unweighted UniFrac distance (Figure 2B) showed separation of group N from group H [Table 7, Adonis: *P* < 0.05 (N vs. H)], whereas PCoA based on binary Jaccard distance (Figure 2A), unweighted UniFrac distance (Figure 2B), and Bray–Curtis distance (Figure 2C) showed that group L was separated from group H [Table 7, Adonis: *P* < 0.05 (L vs. H)].

**Table 7:** Adonis analysis of the gut bacterial communities of winter fur-growing raccoon dogs.

**Group**	**Binary Jaccard**		**Unweighted UniFrac**		**Bray–Curtis**	
	**R** ^2^	***P*** **-value**	**R** ^2^	***P*** **-value**	**R** ^2^	***P*** **-value**
N vs. L	0.110	0.028	0.099	0.277	0.083	0.470
N vs. H	0.243	0.003	0.149	0.006	0.130	0.076
L vs. H	0.278	0.002	0.202	0.003	0.160	0.023

N group, 0 CFU/g *Cyberlindnera jadinii*; L group, 1 × 10^9^ CFU/g *Cyberlindnera jadinii*; H group, 5 × 10^9^ CFU/g *Cyberlindnera jadinii*.

At the phylum level, the top 10 bacteria in relative abundance were identified, and the results indicated that Bacteroidota (N = 49.55 ± 5.49%, L = 48.90 ± 4.19%, H = 50.36 ± 7.19%), Firmicutes (N = 40.99 ± 5.56%, L = 41.93 ± 4.02%, H = 39.24 ± 7.02%), unidentified_Bacteria (N = 4.19 ± 0.34%, L = 3.58 ± 0.49%, H = 4.28 ± 0.12%), and Proteobacteria (N = 3.09 ± 0.73%, L = 2.99 ± 0.74%, H = 2.25 ± 0.87%) were the most abundant bacteria in all three groups (Figure 2D). At the genus level, *Prevotella_9* was the dominant genus in all three groups (N = 32.91 ± 4.36%, L = 30.75 ± 5.09%, H = 36.54 ± 5.91%). While *Alloprevotella* (7.65 ± 0.94%), *Prevotella* (4.50 ± 0.68%), *Faecalibacterium* (3.19 ± 0.62%), and *Streptococcus* (2.93 ± 1.90%) were the most abundant genera in group N. *Alloprevotella* (8.51 ± 1.08%), *Faecalibacterium* (6.41 ± 2.51%), *Prevotella* (5.32 ± 0.55%), and *Dialister* (4.46 ± 1.56%) were the most abundant genera in group L, and *Megamonas* (6.43 ± 5.96%), *Alloprevotella* (6.38 ± 2.43%), *Streptococcus* (4.92 ± 2.36%), and *Megasphaera* (3.70 ± 1.93%) were the most abundant genera in group H (Figure 2E).

**Figure 3:**
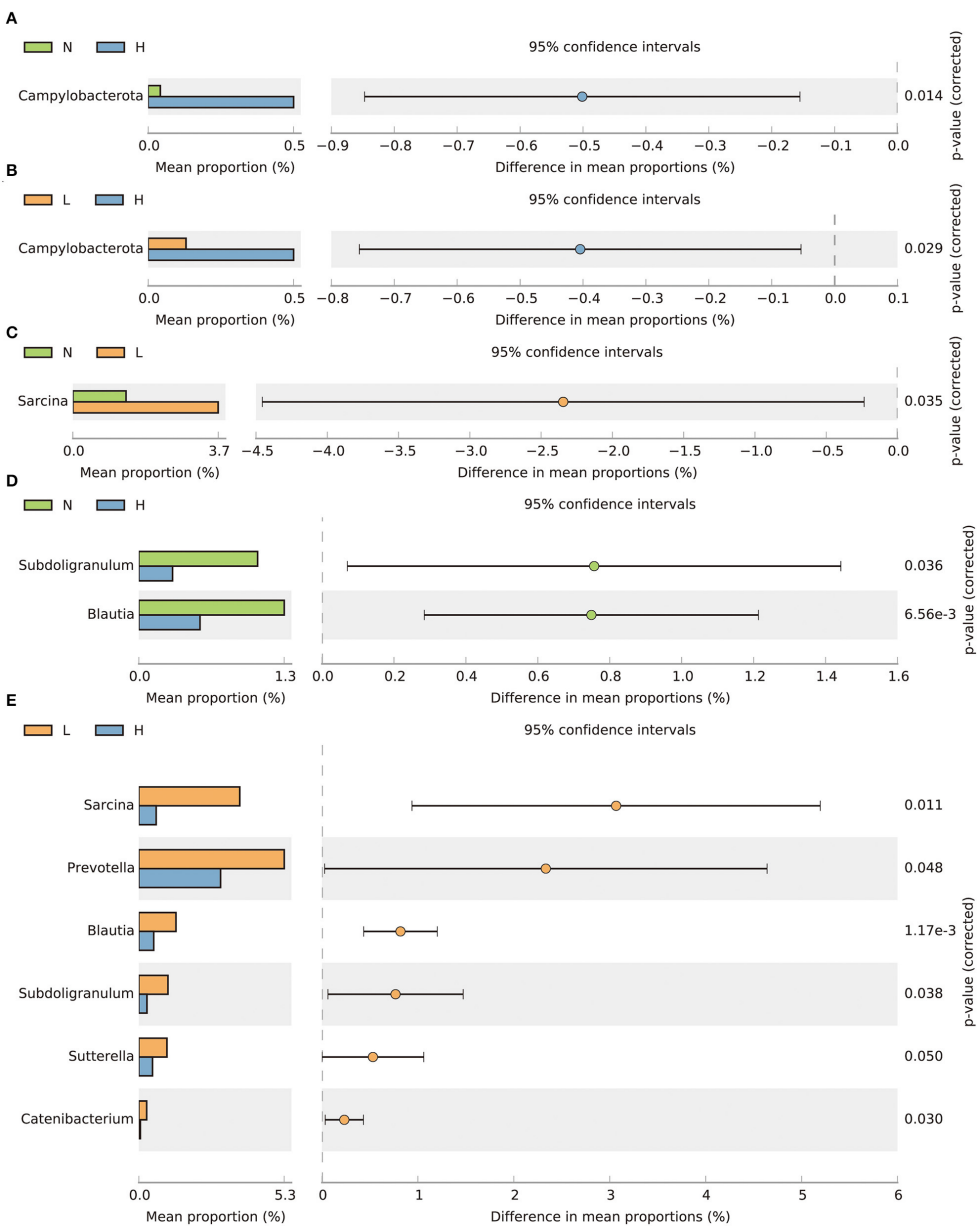
T-test bar plots showing differences in relative abundance of bacteria at the phylum **(A, B)** and genus **(C–E)** levels. N group, 0 CFU/g *Cyberlindnera jadinii*; L group, 1 × 10^9^ CFU/g *Cyberlindnera jadinii*; H group, 5 × 10^9^ CFU/g *Cyberlindnera jadinii*.

By comparing the differences in the relative abundance of bacteria within the phylum, we found that the relative abundance of the Campylobacterota phylum was decreased in groups N and L compared with group H (*P* < 0.05) (Figures 3A, B). At the genus level, the relative abundance of *Sarcina* was increased in group L compared to group N (*P* < 0.05) (Figure 3C). The relative abundance of *Subdoligranulum* and *Blautia* was increased in group N compared with group H (*P* < 0.05) (Figure 3D). In addition, the relative abundance of *Sarcina, Prevotella, Subdoligranulum, Blautia, Sutterella* and *Catenibacterium* in group L were all increased compared with group H (*P* < 0.05) (Figure 3E).

## Discussion

*Cyberlindnera jadinii* can improve growth performance, reduce the diarrhea rate, improve intestinal health, and increase the diversity and abundance of cecal microflora in weaned piglets (15). As shown in our previous study (14), dietary supplementation of *Cyberlindnera jadinii* can increase ADG and decrease F/G in growing raccoon dogs. In the present study, we found that *Cyberlindnera jadinii* could enhance ADG and decrease F/G in winter-growing raccoon dog, and dietary supplementation of *Cyberlindnera jadinii* at 5 × 10^9^ CFU/g resulted in the highest ADG and the lowest F/G. In addition, our results also showed that *Cyberlindnera jadinii* supplementation had no significant effect on the digestibility of dry matter, ether extract and crude protein of raccoon dogs. Indeed, a previous study indicates that dietary supplementation of *Cyberlindnera jadinii* have no obvious effects on the *in vitro* dry matter digestibility of dairy cow (6). However, the dietary supplementation of *Cyberlindnera jadinii* to Hu sheep seems to moderately improve the digestibility of CP (7). This different result might be explained by the experimental animals, appropriate doses of yeast and time of feeding. Likewise, we also observed that *Cyberlindnera jadinii* supplementation had no significant effect on nitrogen metabolism in winter-growing raccoon dogs. Only preliminary studies showed that addition of yeast to feed reduces urinary nitrogen excretion and improve nitrogen deposition and BV in minks (25). This might be because yeast cells are rich in proteins, fats, enzymes, and some coordination factors (26). These factors may lead to different effects and mechanisms of yeast products on the absorption and utilization of nutrients in the digestive tract of animals, but the specific reasons need further study.

Excessive production of free radicals can directly damage proteins, lipids, and nucleic acids, leading to cell death, and consumption of dietary antioxidants is believed to help alleviate oxidative damage and the risk of related diseases, such as cardiovascular disease, cancer, diabetes, and Alzheimer's disease (27, 28). In the present study, *Cyberlindnera jadinii* supplementation was found to increase SOD activity, and 5 × 10^9^ CFU/g *Cyberlindnera jadinii* also increased T-AOC activity. SOD seems to play a fundamental antioxidant role in the detoxification from reactive oxygen species by dismutating superoxide radical anion to oxygen and hydrogen peroxide (29). Increased SOD activity thus plays a crucial role in the natural antioxidant defense system (30). With the improvement of SOD activity, the ability to scavenge oxygen free radicals is enhanced, thereby enhancing the antioxidant capacity (31). T-AOC is the total capacity of various antioxidants to scavenge oxygen free radicals in both enzymatic and non-enzymatic systems (32) and acts as a potent quencher and scavenger of many free radicals (33).

It has been well known that GLU is the most direct source of energy for animals. We found that *Cyberlindnera jadinii* decreased serum GLU concentrations, indicating that *Cyberlindnera jadinii* can promote the efficient use of GLU in winter fur-growing raccoon dogs. Also, oxygen free radicals may damage antioxidant defense system due to hyperglycemia (34). Combined with the antioxidant index results of this study, it seems to suggest that *Cyberlindnera jadinii* may lower serum glucose by scavenging oxygen free radicals. The extended significance of these results may be that adding *Cyberlindnera jadinii* to the diet is beneficial to the stability of blood glucose levels in canine animals, and has a more positive effect on the health of canine animals. In addition, our results showed that *Cyberlindnera jadinii* improved IgA, IgG and IgM levels in winter-growing raccoon dogs. The reason may be that mannan oligosaccharides and 1,3/1,6 β-D-glucans, the two major components of the yeast cell walls, can modulate immunity (35–37). Our results showed that *Cyberlindnera jadinii* supplementation improved the antioxidant activities and immunity in winter-growing raccoon dogs, and that supplementation with 5 × 10^9^ CFU/g *Cyberlindnera jadinii* may be more beneficial.

Diet can alter the intestinal microbiome, which in turn can exhibit a profound impact on overall health (38). In this study, there were significant differences in the microbial community composition of the three groups of winter-growing raccoon dogs, as shown by the PCoA analysis. At the phylum level, the gut microbiota of the winter-growing raccoon dogs were dominated by representative sequences of Bacteroidota, Firmicutes, unidentified_Bacteria, and Proteobacteria. This is different from our previous study (14). The reason may be due to the differences in the living environment and growth stages of the animals. Interestingly, the relative abundance of Firmicutes and Bacteroides in the intestine of winter-growing raccoon dogs accounted for more than 90%. Interestingly, the Firmicutes phyla was found to be enriched in genes related to energy metabolism and material breakdown (39, 40), and the Bacteroidetes was associated with the degradation of proteins and carbohydrates (41, 42). Bacteroidetes are also deeply involved in nutrient metabolism, including carbohydrate and polysaccharide fermentation, and steroid metabolism, and are essential for normal physiological function of the intestine (43). Therefore, it indicates that winter-growing raccoon dogs may need to use more nutrients to meet their own needs. Moreover, the relative abundance of Campylobacterota was significantly increased in group H. Notably, Campylobacterot are recognized as important pathogens: half of the human population is colonized with the ulcer-causing stomach bacterium *Helicobacter pylori*, and *Campylobacter jejuni* is considered the leading cause of bacterial food-borne gastroenteritis worldwide (44–46). The present findings suggested that the addition of 5 × 10^9^ CFU/g *Cyberlindnera jadinii* might increase the abundance of microbiota related to intestinal infection.

Our results showed that at the genus level, the relative abundances of *Subdoligranulum, Sarcina, Sutterella, Blautia, Prevotella* and *Catenibacteriumn* were significantly increased in group L. The increase of *Subdoligranulum* may be due to the presence of undigested compounds such as fibers (47). Further, *Subdoligranulum* may protect the gut through butyrate production (48). *Sarcina* has been implicated in inflammatory processes, which may be related to its ability to produce butyrate through sugar-fermenting (49–51). The relatively high abundance of *Sarcina* in gut- and mucosa-associated microbiota might contribute to a stronger immune resistance in the small intestine (52). *Sutterella* does not appear to induce substantial inflammation (53), but the ability of *Sutterella* to adhere to intestinal epithelial cells indicates that they may have immunomodulatory roles (54). *Blautia* is a genus of anaerobic bacteria with probiotic characteristics widely found in the feces and intestines of mammals, and has been shown to play a role in inflammatory diseases (55). *Prevotella* favors a diet rich in sugars and complex carbohydrates (56). It has also been found to have the characteristic of decomposing starch and plant polysaccharides and is very good at catabolizing mucin (57–59). *Catenibacteriumn* and *Prevotella* are closely correlated to each other, and are strongly associated with long-term diets rich in carbohydrates but low in protein and animal fat (60). *Prevotella* and *Catenibacteriumn* could improve gut health and nutrient utilization by enhancing the fermentation of fiber to produce short chain fatty acids (SCFAs) (61). Previous studies have confirmed that higher concentrations of SCFAs in the intestine contribute to improved growth performance (62). Therefore, supplementation of *Cyberlindnera jadinii* may improve intestinal health by regulating intestinal microbiota, and this effect was greater when the supplementation level was 1 × 10^9^ CFU/g.

## Conclusions

*Cyberlindnera jadinii* has a certain beneficial probiotic effect on raccoon dogs. The combined analysis in this manuscript demonstrated that two feeding levels of *Cyberlindnera jadinii* (1 × 10^9^ CFU/g or 5 × 10^9^ CFU/g) could improve growth performance, immunity, antioxidant abilities and intestinal microbiota, and lowered blood glucose level in winter fur-growing raccoon dogs. Considering the comprehensive cost, 1 × 10^9^ CFU/g may be the most suitable addition level under the experimental conditions.

## Data availability statement

The datasets presented in this study can be found in online repositories. The names of the repository/repositories and accession number(s) can be found at: https://www.ncbi.nlm.nih.gov/, PRJNA929307.

## Ethics statement

The animal study was reviewed and approved by the Animal Care Committee of Chifeng University and conducted in strict compliance with the Committee's guidelines on animal care. Written informed consent was obtained from the owners for the participation of their animals in this study.

## Author contributions

DZ: conceptualization, formal analysis, and writing—original draft. HL and HZ: funding acquisition, methodology, project administration, and writing—review and editing. DZ and YW: data curation, investigation, and supervision. KL, CM, and BZ: data curation and sample collection. All authors have read and approved the final manuscript.
